# Two-stage management of Amyand’s hernia with extensive inguinal abscess: laparoscopic appendiceal transection followed by laparoscopic hernia repair: a case report

**DOI:** 10.1097/RC9.0000000000000220

**Published:** 2026-02-09

**Authors:** Yasunori Shirakawa, Hirofumi Hirao, Hiroto Nishino, Tomoaki Yoh, Shinichi Fujita, Etsuro Hatano

**Affiliations:** a Department of Surgery, Graduate School of Medicine, Kyoto University, Kyoto, Japan; b Department of General Surgery, Tango Central Hospital, Kyoto, Japan

**Keywords:** Amyand’s hernia, inguinal abscess, perforated appendicitis, two-stage management

## Abstract

**Introduction:**

Amyand’s hernia is a rare pathological condition characterized by the presence of the appendix in an inguinal hernia sac. In some cases, it is accompanied by perforated appendicitis, posing a challenging clinical scenario in which the surgeon must address both appendicitis with abdominal sepsis and hernia repair.

**Case presentation:**

A 78-year-old man presented to our hospital with a bulge in the right groin and was diagnosed with Amyand’s hernia with an extensive inguinal abscess on computed tomography. The patient underwent emergency laparoscopic appendiceal transection and groin percutaneous abscess drainage for perforated appendicitis within an inguinal hernia with a groin abscess. The patient was uneventfully discharged on postoperative day 10. Several months later, transabdominal preperitoneal repair with mesh for bilateral inguinal hernia and removal of the scarred residual appendix were performed as second-stage management.

**Discussion:**

Infection control should be prioritized in cases of perforated appendicitis with abscess formation. In cases with no or minimal inflammation, concurrent hernia repair with mesh and appendectomy may be feasible. However, considering the risk of infection, elective hernia repair with a mesh is the preferred approach after inflammation subsides.

**Conclusion:**

We highlighted the importance of individualized surgical management of Amyand’s hernia with severe inflammatory pathology.

## Introduction

Amyand’s hernia is a rare type of inguinal hernia that contains the appendix in the hernia sac, accounting for less than 1% of all inguinal hernias. Approximately half of the patients with Amyand’s hernia present with a normal appendix, another half with acute appendicitis (typically without abdominal sepsis), and a few with abdominal sepsis^[^1^]^. In cases of Amyand’s hernia with strangulation or perforated appendicitis with abscesses, emergency surgical intervention may be warranted, given that Amyand’s hernia is a serious condition associated with a non-negligible mortality rate^[^1,2^]^. Losanoff-Basson classification standardizes the surgical management of Amyand’s hernia based on factors such as the presence of appendicitis and intra-abdominal or abdominal wall sepsis^[^3^]^. However, treatment strategies should be tailored to individual patients, and classification-based treatment strategies should be updated. While several cases of Amyand’s hernia with a risk of infection have been reported, cases with extensive inguinal abscesses are limited.

HIGHLIGHTS

- Amyand’s hernia is a rare type of inguinal hernia that contains the appendix in the hernia sac.
- The management of amyand’s hernia with the risk of infection should be discussed on a case-by-case basis.
- Non-mesh repair is recommended for an inflamed or perforated appendix to reduce the risk of wound or mesh infection.
- In our case, mesh hernia repair was postponed because of the risk of infection, and only laparoscopic appendiceal transection and abscess drainage were initially performed.
- Two-stage hernia mesh repair may be preferable for infection control in cases of Amyand’s hernia accompanied by perforated appendicitis with abscess formation.

Here, we present a case of Amyand’s hernia with an extensive inguinal abscess that was successfully managed with two-stage surgery. This case report was prepared in accordance with the SCARE checklist^[^4^]^.

## Case presentation

**Figure 1.**
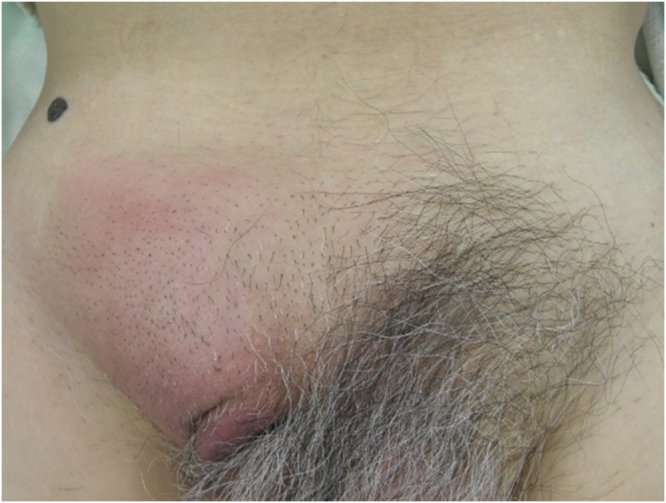
Right groin finding. A fist-sized bulge accompanied by erythema and tenderness of the skin.

**Figure 2.**
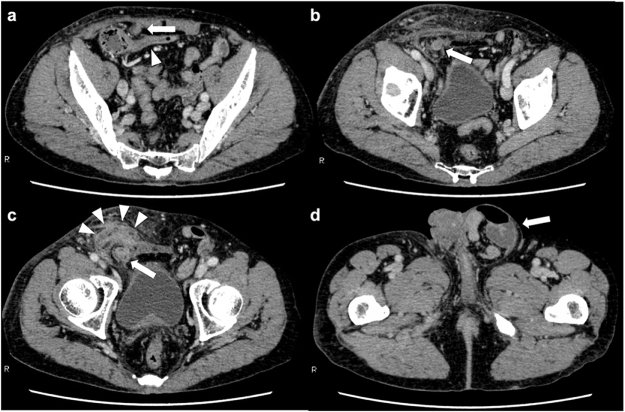
Axial computed tomography scan of the abdomen before the first surgery. (a and b) Right Amyand’s hernia containing the appendix (arrows) contiguous with the terminal ileum (arrowhead). (c) Right inguinal hernia with multiple abscesses (arrowheads). (d) Left inguinal hernia containing a part of small intestine (arrow).

**Figure 3.**
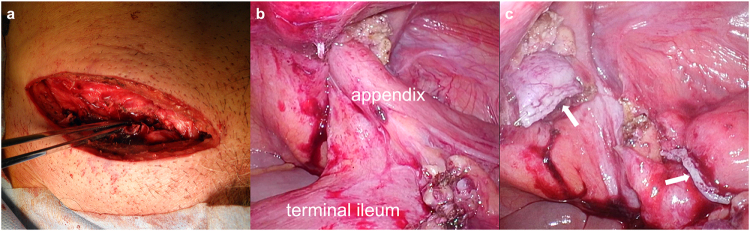
Intraoperative findings during the first surgery. (a) Severe inflammation made it difficult to identify the anatomical structures from the right inguinal incision. (b) Laparoscopic findings of the incarcerated appendix in the right inguinal ring. (c) Laparoscopic findings of the dissected appendix (arrow).

**Figure 4.**
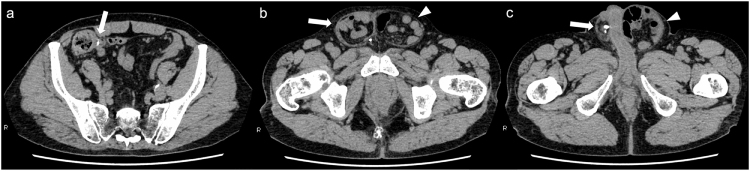
Axial computed tomography scan of the abdomen before the second surgery. (a) Appendiceal root dissected during the first surgery (arrow). (b) Right inguinal hernia (arrow) and left inguinal hernia (arrowhead). (c) Right inguinal hernia containing the appendiceal remnant from the first surgery (arrow) and left inguinal hernia (arrowhead).

**Figure 5.**
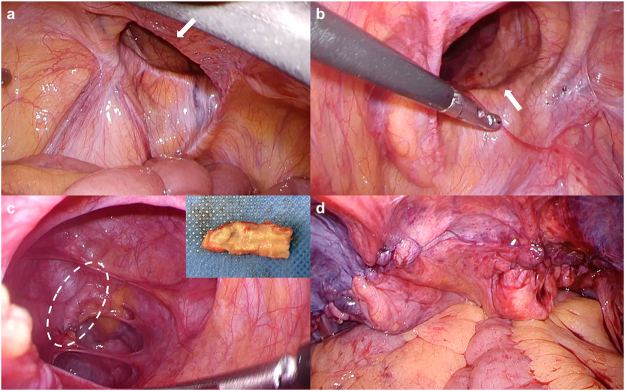
Laparoscopic findings during the second surgery. (a) Left inguinal hernia orifice (arrow). (b) Right inguinal hernia orifice (arrow). (c) The atrophied appendix (circle) was removed (inset). (d) The bilateral hernia orifices were closed.

A man in his 70s was aware of a bulge and pain in the right inguinal region and was treated with antibiotics by a local doctor and made a mild recovery. He presented to a local clinic because of increasing groin pain and was referred to our hospital because of a suspected incarcerated right inguinal hernia. Upon presentation, the patient had a fist-sized bulge in the right inguinal region, accompanied by erythema and tenderness of the skin (Fig. 1). No other significant findings were observed on physical examination. Abdominal computed tomography (CT) revealed a continuous tubular structure from the ileocecal area, distinct from the cecum and terminal ileum, prolapsing into the right inguinal hernia sac. The tip of the structure was indistinct and surrounded by multiple abscesses with a marginal contrast effect in the sac (Fig. 2a–c). Simultaneously, CT revealed a part of the small intestine in the left groin (Fig. 2d). The patient was diagnosed with right Amyand’s hernia and left inguinal hernia. Amyand’s hernia was accompanied by suspected appendiceal perforation; therefore, emergency surgery was performed on the same day. After an incision was made in the right inguinal region, abscess drainage was performed. Initially, appendectomy and non-mesh hernia repair were planned. However, severe inflammation of the inguinal region made it difficult to identify the anatomical structures (Fig. 3a), and the procedure was converted from open to laparoscopic surgery. The right inguinal hernia contained the appendix and mesenteric appendages (Fig. 3b), and hernia reduction from the abdominal cavity was difficult, probably because of the severe edema of the surrounding tissues. The mesoappendix was dissected using a laparoscopic coagulating shear, and the appendiceal root was dissected using an Endo GIA 45 mm (Fig. 3c). The appendix was difficult to recognize through the anterior skin incision and was left dissected in anticipation of sclerosis due to scar atrophy. Mesh hernia repair was postponed because of the risk of infection. A drainage tube was placed under the right inguinal incision, and the operation was completed. The postoperative course was uneventful, with no infectious complications, such as abscesses or wound infections. The drain was removed on postoperative day (POD) 5, and the patient was discharged on POD 10. As no further problems arose and the patient did not request surgery, the patient was kept under observation, although the patient had a large left inguinal hernia. However, 8 months after surgery, abdominal CT revealed the recurrence of the right inguinal hernia and the already recognized left inguinal hernia (Fig. 4a-c); therefore, laparoscopic hernia repair was planned with sufficient informed consent. During surgery, bilateral inguinal hernia orifices were observed (Fig. 5a, b). Under general anesthesia, three trocars were inserted: a 12-mm umbilical port for the camera and two 5-mm working ports in the lower abdomen. The peritoneum was incised transversely approximately 2–3 cm above the hernia defect to create a peritoneal flap. The atrophied appendix was removed en bloc with the tip of the hernia sac (Fig. 5c). The preperitoneal spaces were dissected bilaterally to expose the hernia sacs and the surrounding anatomical landmarks, including the inferior epigastric vessels, Cooper’s ligament, and the spermatic cord structures. Lightweight meshes (3DMax™ Mesh, Large, ref: 0115321, BD) were placed on each side to sufficiently cover the bilateral myopectineal orifices, ensuring adequate coverage of the direct, indirect, and femoral defects (Fig. 5d). Each mesh was fixed to Cooper’s ligament and the anterior abdominal wall using absorbable tacks. The peritoneal flaps were then closed to cover the meshes completely and prevent adhesion to intra-abdominal organs. Pneumoperitoneum was released, trocars were removed, and the incisions were closed in layers. Histopathological examination of the appendix revealed no malignancies. The postoperative course was uneventful, and the patient was discharged on POD 3. Outpatient follow-up was conducted every 3 months, and no infection or hernia recurrence was observed for more than 8 months postoperatively.

## Discussion

In this report, we present a case of Amyand’s hernia with an extensive inguinal abscess that was successfully treated with two-stage surgery. Treatment of Amyand’s hernia with an infection risk requires unusual therapeutic strategies. Infection management was prioritized during the first surgery, followed by hernia repair during the second surgery. According to a report published in 2003, the mortality rate was 14%–30%^[^2^]^, whereas a report in 2021 documented a rate of 1.8%^[^1^]^. This reduction is presumed to be attributable to factors such as the availability of earlier imaging-based diagnoses, advancements in surgical techniques, and improvements in postoperative management. Nevertheless, Amyand’s hernia represents a significant surgical challenge and is associated with a high mortality rate.

The incidence of Amyand’s hernia is reported to be approximately 0.14%–1.3% of inguinal hernias, whereas the incidence of Amyand’s hernia with appendicitis is lower, estimated to be 0.07%–0.13%^[^5^]^. Losanoff-Basson classified Amyand’s hernia into four types: type 1, normal appendix; type 2, acute appendicitis with no abdominal sepsis; type 3, acute appendicitis with abdominal sepsis; and type 4, acute appendicitis with abdominal pathology^[^3^]^. The current case was classified as type 2 because the abscess was not located within the peritoneal cavity. Based on the Losanoff-Basson Classification, type 2 cases are recommended to undergo appendectomy through hernia and hernioplasty without mesh. Therefore, we initially attempted hernia repair without mesh using an open approach; however, the distorted anatomy due to the extensive abscess precluded reliable identification of structures essential for definitive non-mesh repair, necessitating second-stage mesh repair. Among the three main methods of treating inguinal hernias (open, laparoscopic, and robotic), open and laparoscopic approaches are the most common^[^6^]^. Despite its significantly lower prevalence, the comparable safety and postoperative outcomes of robotic repair relative to laparoscopy suggest an anticipated increase in clinical adoption^[^7^]^. The surgical approach for Amyand’s hernia has predominantly been open^[^8^]^, which can achieve both tissue repair and appendectomy simultaneously through an inguinal incision^[^9^]^. However, the number of reports on laparoscopic management has increased in recent years^[^10^]^. The advantages of laparoscopic surgery include not only the minimally invasive aspects but also the capability of direct evaluation of the incarcerated contents via laparoscopy. Despite these advantages, laparoscopic inguinal hernia repair requires the use of mesh; hence, open surgery could be a primary treatment option in cases where there is concern about infection.

A recent review of inguinal hernia studies comparing mesh hernia repair with non-mesh repair demonstrated the superiority of mesh repair in preventing hernia recurrence^[^11^]^. Although successful simultaneous treatment of appendectomy and hernia mesh repair without complications has been reported in cases with no or minimal appendicitis, non-mesh repair is recommended for an inflamed or perforated appendix to reduce the risk of wound or mesh infection^[^12^]^. Therefore, we initially chose an anterior approach to treat the hernia without using a mesh. Although the drainage of the abscess cavity was achieved, severe inflammation was observed around the inguinal region, making it challenging to identify landmarks such as the spermatic cord, inguinal canal, inferior epigastric artery, and round ligament, which are essential for a safe operation^[^13^]^. We then changed the surgical approach from open to laparoscopic surgery. Consistent with the preoperative diagnosis, the appendix was incarcerated within the hernia orifice. However, pronounced edema in the surrounding tissues rendered the identification of the inferior epigastric artery, spermatic cord, and related structures challenging. To avoid secondary injury, the incarcerated appendix was not forcibly reduced. Instead, only the minimum intervention of transecting the base of the appendix to interrupt the communication with the cecum was performed to prevent fecal fistula, while definitive hernia repair was postponed for a second surgery. Indeed, cases of enterocutaneous fistula have been reported, although the exact incidence remains unknown^[^14,15^]^.

Only six cases of two-stage surgery for Amyand’s hernia have been reported to date^[^9,16–20^]^ (Table 1). Five patients underwent appendectomy followed by hernia repair, and one patient underwent abscess drainage followed by appendectomy and hernia repair. The main reason for the two-stage surgery was to reduce the risk of infection during the first stage. The details of the second stage of hernia repair are described in four cases, all of which used mesh. Although two-stage hernia mesh repair is preferable for infection control, one-stage tissue repair remains an alternative. This case is unique in that technical factors, such as difficulty in identifying anatomy, limited the one-stage hernia repair. Consequently, second-stage mesh hernia repair following infection control ultimately minimized the risks of both mesh-related infection and hernia recurrence.

**Table 1:** Cases of Amyand’s hernia managed by two-stage treatment.

Case	Year	First author	Age, sex	Preoperative diagnosis	Appendix perforation	Abscess	First treatment	Second treatment	Mesh	SSI	Reason for two-stage treatment
1	2018	Akaishi	70, M	Appendicitis with right inguinal hernia	Yes	Yes	Laparoscopic appendectomy	Open hernia repair (Direct Kugel)	Yes	No	To avoid postoperative SSI
2	2023	Radboy	48, M	Amyand’s hernia	No	No	Laparoscopic appendectomy	Hernia repair (NR for details)	Yes	No	To avoid both wound and mesh infection
3	2024	Chiba	82, M	Amyand’s hernia	Yes	Yes	Laparoscopic appendectomy with abscess drainage	Open hernia repair (Lichtenstein)	Yes	No	To avoid the risk of postoperative mesh and wound infection
4	2024	Chagam	76, M	Concurrent inguinal hernia and appendicitis	Yes	Yes	CT-guided abscess drainage	Laparoscopic appendectomy with drainage and primary hernia repair	Yes	No	NR
5	2024	Choi	71, M	Strangulated right inguinal hernia	No	Yes	Laparoscopic appendicectomy	TAPP inguinal hernia repair	Yes	No	To perform hernia repair after local inflammation has resolved
6	2025	Burgos	31, M	Amyand’s hernia	Yes	Yes	Laparoscopic appendicectomy with JP drain placement	Hernia repair (NR for details)	NR	No	To minimize the risk of SSI and recurrence
Present case	2025	Shirakawa	70s, M	Amyand’s hernia	Yes	Yes	Laparoscopic appendiceal transection and percutaneous abscess drainage	TAPP inguinal hernia repair	Yes	No	To avoid infection Technical difficulty of one-stage non-mesh hernia repair

M, male; F, female; CT, computed tomography; SSI, surgical site infection; TAPP, transabdominal preperitoneal; NR, not reported.

## Conclusion

We present a case of Amyand’s hernia with an extensive inguinal abscess that was successfully managed with two-stage surgery. Given that the management of Amyand’s hernia with the risk of infection should be discussed on a case-by-case basis, further cases are warranted.

## Data Availability

The data supporting the findings of this study are available from the corresponding author upon reasonable request.

## Notes

Not commissioned.

## References

[1] Manatakis DK Tasis N Antonopoulou MI. Revisiting Amyand’s hernia: a 20-year systematic review. World J Surg 2021;45:1763–70. 33598722 10.1007/s00268-021-05983-y

[2] D’Alia C Lo Schiavo MG Tonante A. Amyand’s hernia: case report and review of the literature. Hernia 2003;7:89–91. 12820031 10.1007/s10029-002-0098-5

[3] Losanoff JE Basson MD. Amyand Hernia: What Lies Beneath–A Proposed Classification Scheme to Determine Management. Am Surg 2007;73:1288–90. 18186392

[4] Kerwan A Al-Jabir A Mathew G. Revised Surgical CAse REport (SCARE) guideline: An update for the age of Artificial Intelligence. Prem J Sci 2025;10:100079.

[5] Ivanschuk G Cesmebasi A Sorenson EP. Amyand’s hernia: A review. Med Sci Monit 2014;20:140–46. 24473371 10.12659/MSM.889873 PMC3915004

[6] Nanayakkara KDL Viswanath NG Wilson M. An international survey of 1014 hernia surgeons: outcome of GLACIER (Global Practice of Inguinal Hernia Repair) study. Hernia 2023;27:1235–43. 37310493 10.1007/s10029-023-02818-8

[7] Solaini L Cavaliere D Avanzolini A. Robotic versus laparoscopic inguinal hernia repair: an updated systematic review and meta-analysis. J Robot Surg 2022;16:775–81. 34609697 10.1007/s11701-021-01312-6 PMC9314304

[8] Towfigh S. Inguinal Hernia: Four Open Approaches. Surg Clin North Am 2018;98:623–36. 29754626 10.1016/j.suc.2018.02.004

[9] Malik MIK Abbas J Shuttleworth P. Perforated necrotic appendix in Amyand’s hernia treated with appendicectomy and simple suture repair of the inguinal hernia. BMJ Case Rep 2019;12:e231765. 10.1136/bcr-2019-231765 PMC6855868 31694829

[10] Choi CC Taylor D Tokhi A. Amyand’s hernia with concurrent appendicitis: A case of interval laparoscopic herniorrhaphy and literature review. Int J Surg Case Rep 2024;118:109601. 38608522 10.1016/j.ijscr.2024.109601 PMC11017271

[11] Lockhart K Dunn D Teo S. Mesh versus non-mesh for inguinal and femoral hernia repair. Cochrane Database Syst Rev 2018;9:CD011517. 30209805 10.1002/14651858.CD011517.pub2 PMC6513260

[12] Bratu D Mihetiu A Sandu A. Controversies regarding mesh utilisation and the attitude towards the appendix in Amyand’s hernia—a systematic review. Diagnostics (Basel) 2023;13:3534. 38066775 10.3390/diagnostics13233534 PMC10706417

[13] Miller HJ. Inguinal Hernia: Mastering the Anatomy. Surg Clin North Am 2018;98:607–21. 29754625 10.1016/j.suc.2018.02.005

[14] Yagnik VD. Amyand’s hernia. J Indian Assoc Pediatr Surg 2012;17:88. 10.4103/0971-9261.93978 PMC3326834 22529560

[15] Panagidis A Sinopidis X Zachos K. Neonatal perforated Amyand’s hernia presenting as an enterocutaneous scrotal fistula. Asian J Surg 2015;38:177–79. 24751296 10.1016/j.asjsur.2014.03.001

[16] Akaishi R Nishimura R Naoshima K. Amyand’s hernia complicated with appendix perforation treated by two-stage surgery consisting of laparoscopic appendectomy followed by elective inguinal hernioplasty: A case report. Int J Surg Case Rep 2018;47:11–13. 29702463 10.1016/j.ijscr.2018.04.019 PMC5994868

[17] Radboy M Kalantari ME Einafshar N. Amyand hernia as a rare cause of abdominal pain: A case report and literature review. Clin Case Rep 2023;11:e7929. 37780933 10.1002/ccr3.7929 PMC10533375

[18] Chiba Y Usuda D Yamamoto T. A Case of Amyand’s Hernia With Abscess Managed by Two-Stage Surgery: Elective Hernia Mesh Repair Following Emergency Laparoscopic Appendectomy. Cureus 2024;16:e68486. 39364462 10.7759/cureus.68486 PMC11446888

[19] Chagam L Modi R Toub F. Amyand’s Hernia: A Rare Case Study of Perforated Appendicitis in an Inguinal Hernia. Cureus 2024;16:e56898. 38659534 10.7759/cureus.56898 PMC11042761

[20] Miranda Burgos L Thomas A Fluss W. Management of Perforated Appendicitis in Amyand’s Hernia: A Multidisciplinary Approach to Complex Postoperative Complications. Cureus 2025;17:e81498. 40308412 10.7759/cureus.81498 PMC12042590

